# Radix Astragali and Radix Angelicae Sinensis in the Treatment of Idiopathic Pulmonary Fibrosis: A Systematic Review and Meta-analysis

**DOI:** 10.3389/fphar.2020.00415

**Published:** 2020-04-30

**Authors:** Yufeng Zhang, Lina Gu, Qingqing Xia, Lijun Tian, Jia Qi, Mengshu Cao

**Affiliations:** ^1^Department of Respiratory Medicine, Jiangyin Hospital of Traditional Chinese Medicine, Jiangyin Hospital Affiliated to Nanjing University of Chinese Medicine, Jiangyin, China; ^2^Department of Pulmonary and Critical Care Medicine, Nanjing Drum Tower Hospital Clinical College of Traditional Chinese and Western Medicine, Nanjing University of Chinese Medicine, Nanjing, China; ^3^Department of Pulmonary and Critical Care Medicine, Nanjing Drum Tower Hospital, The Affiliated Hospital of Nanjing University Medical School, Nanjing, China; ^4^Department of Critical Care Medicine, Nantong Third People's Hospital, Nantong University, Nantong, China; ^5^Department of Pharmacy, Xin Hua Hospital Affiliated to Shanghai Jiao Tong University School of Medicine, Shanghai, China

**Keywords:** Radix Astragali, Radix Angelicae Sinensis, idiopathic pulmonary fibrosis, treatment, systematic review, meta-analysis

## Abstract

**Introduction:**

There are many clinical studies in the treatment of idiopathic pulmonary fibrosis (IPF) with herbal medicine including Astragalus mongholicus Bunge, Radix Astragali (RA) and Angelica sinensis (Oliv.) Diels, Radix Angelicae Sinensis (RAS). These have obtained good curative effect. There is no systematic evaluation on the clinical efficacy of RA and RAS in patients with IPF. The aim of this systematic review and meta-analysis was to critically evaluate the current evidence of efficacy and safety of RA and RAS in IPF.

**Methods:**

We searched the primary database for randomized controlled trial (RCT) of RA and RAS treating IPF. We assessed the quality of included studies using the Jadad rating scale and referred to the Cochrane Reviewer's Handbook for guidelines to assess the risk of bias. We extracted the main outcomes of included RCTs and a meta-analysis was conducted using the Cochrane Collaboration's RevMan5.3 software.

**Results:**

Seventeen eligible RCTs were identified and made a systematic review and meta-analysis. Risk of bias and quality of included RCTs were carried out. The results of meta-analysis showed that total effective rate and traditional Chinese medicine syndrome effective rate were statistically significantly higher in the experimental group than the control group, main pulmonary function index, six minute walking distance and Borg scale questionnaire score were statistically significantly better in the experimental group than the control group and incidence of adverse reactions was statistically significantly lower in the experimental group than the control group.

**Conclusion:**

RA and RAS are effective and safe in the treatment of IPF, which is beneficial to pulmonary function and exercise tolerance of these patients.

## Introduction

Idiopathic pulmonary fibrosis (IPF) is a type of interstitial lung disease characterized as chronic, progressive and fibrotic, and its clinical manifestation is progressive aggravation of dyspnea, restrictive ventilation dysfunction and gas-exchange disorder, hypoxemia and even respiratory failure (Cao et al., 2019). The chest high-resolution CT (HRCT) or lung histology of IPF is characteristic of usual interstitial pneumonia (UIP) (Raghu et al., 2018). IPF is a rare disease, which is prone to the elderly. In Europe and North America, the incidence of IPF is about 2.8-9.3 per 100,000, and the epidemiological data in China is not much, but the incidence of IPF in recent years has increased significantly (Navaratnam et al., 2011; Huang et al., 2013; Hutchinson et al., 2015). IPF is currently incurable, and the clinical purpose is to delay the deterioration of lung function, improve the quality of life and delay the progress of the disease. At present, western medicine, such as antifibrotic drugs, has certain curative effect in the treatment of IPF, but due to the high price and some side effects, it is restricted in patient use (Lee et al., 2013). In recent years, the position of traditional Chinese medicine (TCM) in the treatment of IPF is becoming more and more important, and the clinical research and meta-analyses have shown that the herbal medicine treating IPF could improve the clinical symptoms, delay the reduction of the lung function, and improve the quality of life of the patients (Yu et al., 2016; Chen et al., 2019; Wu et al., 2019). Many experiments have shown that the herbal medicine has the effects of improving the pathological and pulmonary function of bleomycin-induced IPF rats (Chen et al., 2016; Yu et al., 2018).

Herbal medicine is the main treatment of TCM, the collocation of monarch herbs and minister herbs is adjusted according to the common pathogenesis of patients with further prescription to adapt to the different pathogenesis of IPF. DangGuiBuXue Decoction has the history of nearly 800 years, and is composed of two commonly used Chinese herbal medicines of Astragalus mongholicus Bunge, Radix Astragali (RA) and Angelica sinensis (Oliv.) Diels, Radix Angelicae Sinensis (RAS), and has the effects of benefiting vital energy and promoting blood circulation (Shi et al., 2019). At present, based on the association rules of the literature, the treatment of IPF with TCM is mainly related to benefiting vital energy and promoting blood circulation, among which RA and RAS are the most common herbs for invigorating qi and activating blood (Ren, 2017; Huang et al., 2018). There are many experiments on the treatment of IPF, which manifest RA and RAS can improve pulmonary fibrosis in animal model (Liu, 2009; Li et al., 2015). Our recent research shows that RA and RAS in the treatment of IPF through the multi-target and multi-pathway were systematically discussed, which plays an important role in the clinical application (Zhang et al., 2019).

At present, there are few clinical studies on the treatment of IPF with RA and RAS only, but many clinical studies on the treatment of IPF used herbal medicine included RA and RAS as the main components and have obtained good curative effect (Sun, 2005; Wei and Qiang, 2007; Sun et al., 2008). There is no systematic evaluation report on the clinical efficacy of RA and RAS as the main components of herbal medicine in the treatment of IPF. The aim of this systematic review and meta-analysis was to critically evaluate the current evidence of effectiveness and safety on the use of RA and RAS in the treatment for patients with IPF.

## Materials and Methods

### Data Sources and Search Strategy

We searched the main English and Chinese databases from the establishment of the database to October 30, 2019. PUBMED, EMBASE, Science Citation Index (SCI), Cochrane Central Register of Controlled Trials, Chinese Biomedical Literature database (SinoMed), Chinese National Knowledge Infrastructure (CNKI), Wanfang Data and the Chongqing VIP database(CQVIP) were included.

The search term “pulmonary fibrosis” was combined with the following keywords respectively: “Astragali”; “Angelicae”; “DangGuiBuXue Decoction”; “DangGuiBuXue Tang”; “traditional Chinese Medicine”; “Chinese Medicine”; “herbal medicine”. We also searched for these terms in titles and abstracts. When such data were not included in abstracts, if such data existed in the full text, the full-text paper was screened as well. We also checked references and citations of the identified studies manually to include other potentially eligible trials until no additional articles could be identified.

### Inclusion and Exclusion Criteria

Inclusion criteria: (1) The study was designed as a randomized controlled trial (RCT); (2) The participants were in accordance with the diagnosis of IPF, which is in line with the Chinese Medical Association Respiratory Society issued guidelines for diagnosis and treatment or ATS/ERS/JRS/ALAT Clinical Practice Guideline; (3) Herbal medicine included RA and RAS was used in the experimental group; (4) The control group used conventional therapy without TCM therapy; (5) There were clear outcome measures.

Exclusion criteria: (1) Duplicated publications, the earlier published or the one with most complete information was included and the rest were excluded; (2) Animal experiments; (3) Case reports, reviews and abstracts; (4) Lack of data outcome measures to evaluate the effects.

### Quality Assessment and Data Extraction

Using the Jadad rating scale (Higgins et al., 2011) and the Cochrane Reviewer's Handbook for guidelines, the quality and risk of bias of included studies were assessed (Higgins et al., 2011).

The scores were obtained by evaluating a RCT with three items describing randomization (0-2 points), blinding (0-2 points), and dropouts and withdrawals (0-1 points). One point was given for each term if these terms were mentioned in the study. If the method to generate the sequence of randomization or the method of blinding was described and appropriate, then 1 additional point was given, whereas 1 point was deducted if it was inappropriate. The quality scale ranges from 0 to 5 points. Higher scores indicate better reporting. It was divided into low quality less than 3 and high quality greater than or equal to 3 (Jadad et al., 1996).

We used the Cochrane classification of seven criteria to assess the risk of bias, which contained: random sequence generation, allocation concealment, patient blinding, assessor blinding, incomplete outcome data, selective outcome reporting and other risks of bias (Higgins et al., 2011).

Two reviewers independently extracted the information of data, which included: the first author, year of publication, number of patients in each group, major composition of TCM prescriptions, methods of intervention on experimental group and control group and outcomes.

All authors consulted the disagreement about the detail of study until it was resolved by consensus.

### Statistical Analyses

The Cochrane Collaboration's RevMan5.3 software was used for systematic review and meta-analysis. Continuous data were expressed as mean difference (MD) with 95% confidence interval (CI). Dichotomous data were expressed as odds ratio (OR) with 95% CI. A test of heterogeneity was assessed by the Q test (*P* value and *I²*), which describes the percentage of variability in the effect and estimates the contribution of heterogeneity rather than by chance (Higgins and Thompson, 2002; Higgins et al., 2003). A significant Q-statistic (*P* < 0.10) indicated heterogeneity across studies. Studies with an *I²* statistic of less than 50% are considered to have no heterogeneity and those with an *I²* statistic of equal or more than 50% are considered to have heterogeneity. If no significant heterogeneity was detected, the fixed effects model was used as the pooling method; otherwise, the random effect model was considered to be the appropriate choice. We perform the funnel plot to determine publication bias when more than 10 studies are included in a meta-analysis. All reported probabilities (*P* values) were two-sided, and *P*< 0.05 was considered statistically significant.

## Results

### Research Selection

**Figure 1:**
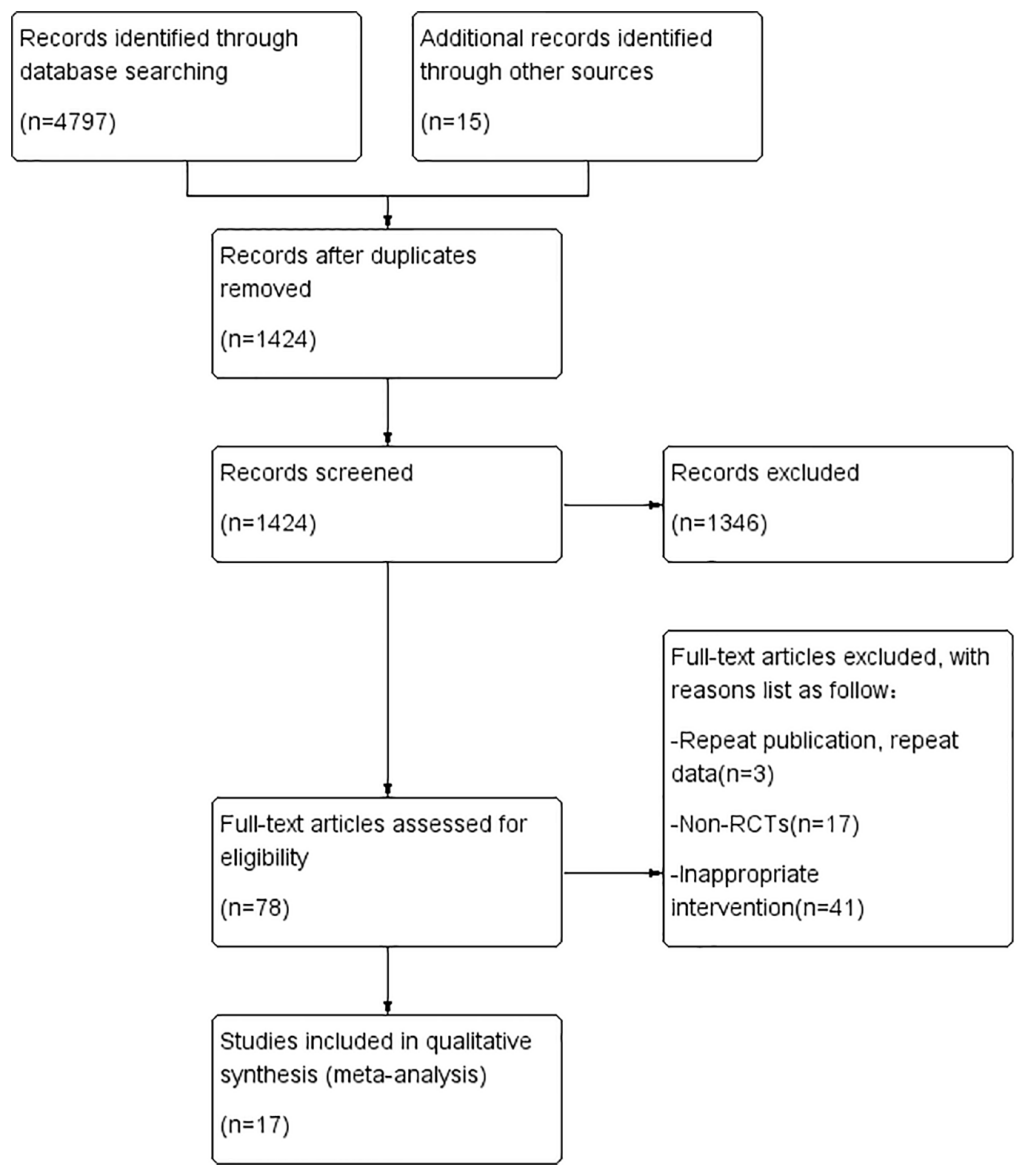
Flow chart of the research selection process.

A total of 4812 studies were retrieved through database searching and other sources. After removing duplication, 1424 studies had been retained. A total of 1346 obviously irrelevant studies were excluded after reading the title and the abstract, another 61 studies were excluded due to various reasons after reading the full text. Seventeen RCTs were included in the systematic evaluation (Sun, 2005; Wei and Qiang, 2007; Sun et al., 2008; Dong, 2010; Yang, 2010; Wang, 2011; Chen et al., 2012; Wu et al., 2012; Meng et al., 2016; Zhao et al., 2016; Jiang, 2017; Deng and Wang, 2018; Ma, 2018; Miao et al., 2018; Yang, 2018; Guo et al., 2019; Peng, 2019). The literature screening process and results are shown in **Figure 1**.

### Description of Included Studies

Seventeen eligible RCTs (Sun, 2005; Wei and Qiang, 2007; Sun et al., 2008; Dong, 2010; Yang, 2010; Wang, 2011; Chen et al., 2012; Wu et al., 2012; Meng et al., 2016; Zhao et al., 2016; Jiang, 2017; Deng and Wang, 2018; Ma, 2018; Miao et al., 2018; Yang, 2018; Guo et al., 2019; Peng, 2019) were identified. Seventeen RCTs were all conducted in China and included 1211 patients. Two studies (Sun, 2005; Guo et al., 2019) were multicenter studies and others were single-center studies. One RCT (Peng, 2019) used the prescription of TCM only included RA and RAS and other RCTs used the prescription of TCM included RA and RAS as the main components. The control group included conventional western medicine treatment (CWMT), while prednisone tablets were used in a number of studies (Sun, 2005; Wei and Qiang, 2007; Sun et al., 2008; Dong, 2010; Yang, 2010; Wang, 2011; Jiang, 2017; Deng and Wang, 2018; Yang, 2018; Peng, 2019); prednisone tablets and cyclophosphamide tablets were used in three studies (Wu et al., 2012; Meng et al., 2016; Miao et al., 2018); acetylcysteine was used in two studies (Zhao et al., 2016; Ma, 2018); one study (Guo et al., 2019) used placebo granules; one study (Chen et al., 2012) only mentioned the use of CWMT. Basic features of included studies are outlined in **Table 1**, the composition of TCM prescriptions used in experimental group of each study are outlined in **Table 2** and the quality control of TCM prescriptions are outlined in **Table 3**.

**Table 1:** Summary of RCTs of RA and RAS for IPF.

Study year[ref]	Country	Sample size (Experimental/Control)	Mean age (years) (Experimental/Control)	Experimental	Control	Duration
Sun XS 2005 (Sun, 2005)	China	60(30/30)	61.10 ± 12.88/66.67 ± 7.70	QiHong Decoction	Prednisone tablet	3 months
Wei GS 2007 (Wei and Qiang, 2007)	China	54(36/18)	40-78(58.4)/38-74(55.2)	CWMT + Prednisone tablet + TongFeiHuoXue Decoction	CWMT + Prednisone tablet	1 month
Sun ZT 2008 (Sun et al., 2008)	China	30(15/15)	56.45 ± 7.88/56.88 ± 9.76	Prednisone tablet + YiQiHuoXueSanJie Basic Prescription	Prednisone tablet	3 months
Dong H 2010 (Dong, 2010)	China	66(33/33)	59.11 ± 11.18/57.7 ± 10.4	Prednisone tablet + KangXianShuFei Granules	Prednisone tablet	3 months
Yang ZJ 2010 (Yang, 2010)	China	40(20/20)	60.4 ± 8.61/60.25 ± 8.72	YiQiYangYin Prescription	Prednisone tablet	3 months
Wang F 2011 (Wang, 2011)	China	28(14/14)	52.3 ± 3.6/54.5 ± 4. 6	Prednisone tablet + KangXianShuFei Chinese Medicine	Prednisone tablet	6 months
Chen P 2012 (Chen et al., 2012)	China	50(25/25)	55-72(63)/53-74(65)	CWMT + HuaXianPoGu Decoction	CWMT	3 months
Wu HS 2012 (Wu et al., 2012)	China	71(36/35)	41-76(62.5)/42-79(63.4)	CWMT + Prednisone tablet + Cyclophosphamide tablet + KangYangHuaXianRuanFei Magical Prescription	CWMT + Prednisone tablet + Cyclophosphamide tablet	6 months
Meng Y 2016 (Meng et al., 2016)	China	80(40/40)	63.52/n.r.	Prednisone tablet + Cyclophosphamide tablet + YiQiYangXue Chinese Medicine Decoction	Prednisone tablet + Cyclophosphamide tablet	2 months
Zhao YD 2016 (Zhao et al., 2016)	China	120(60/60)	55.17 ± 13.13/57.26 ± 10.14	CWMT + N-acetylcysteine tablet + BuFeiHuoXueHuaPi Prescription	CWMT + N-acetylcysteine tablet	12 months
Jiang WZ 2017 (Jiang, 2017)	China	80(40/40)	65.58 ± 3.35/65.62 ± 3.40	CWMT + Prednisone tablet + YiQiHuoTanZhuYu Chinese Medicine Prescription	CWMT + Prednisone tablet	40 days
Miao G 2018 (Miao et al., 2018)	China	80(40/40)	67.4 ± 4.7/66.3 ± 4.8	CWMT + Prednisone tablet + cyclophosphamide + YiQiYangYinSanJieHuaTan Prescription	CWMT + Prednisone tablet + Cyclophosphamide tablet	2 months
Yang QM 2018 (Yang, 2018)	China	82(41/41)	67.16 ± 7.84/67.56 ± 7.14	CWMT + Prednisone tablet + YiQiHuoTanZhuYu Decoction	CWMT + Prednisone tablet	1 month
Ma Q 2018 (Ma, 2018)	China	72(36/36)	67.93 ± 8.49/69.17 ± 7.98	CWMT + Echinocysteine effervescent tablet + BuYangHuanWu Decoction and LiuJunZi Decoction	CWMT + Acetylcysteine effervescent tablet	12 weeks
Deng F 2018 (Deng and Wang, 2018)	China	118(59/59)	64.06 ± 7.82/63.21 ± 7.45	CWMT + Prednisone tablet + HuangQiTaoHong Decoction	CWMT + Prednisone tablet	3 months
Guo SJ 2019 (Guo et al., 2019)	China	130(65/65)	59.45 ± 5.19/58.62 ± 5.02	CWMT + QiZhuKangXian Granules	CWMT + placebo	48 weeks
Peng YF 2019 (Peng, 2019)	China	50(25/25)	58.96 ± 8.73/59.80 ± 9. 34	CWMT + Prednisone tablet + QiGui Prescription	CWMT + Prednisone tablet	12 weeks

RCT, randomized controlled trial; RA, Radix Astragali; RAS, Radix Angelicae Sinensis; IPF, Idiopathic pulmonary fibrosis; CWMT, conventional western medicine treatment; n.r., not reported.

**Table 2:** Composition of TCM prescriptions.

Study year[ref]	TCM prescriptions	Composition of TCM prescriptions		
		Latin name	English name	Chinese name
Sun XS 2005 (Sun, 2005)	QiHong Decoction	**Radix Astragali** **Radix Angelicae Sinensis** Flos Carthami Radix Curcumae Flos Inulae Semen Coicis Radix Platycodi	**Astragalus Root** **Chinese Angelica Root** Safflower Curcuma Tuber Inula Flower Job's Tears Seed Balloon Flower Root	**HuangQi** **DangGui** HongHua YuJin XuanFuHua YiYiRen JieGeng
Wei GS 2007 (Wei and Qiang, 2007)	TongFeiHuoXue Decoction	**Radix Astragali** **Radix Angelicae Sinensis** Flos Lonicerae Radix Salviae Miltiorrhizae Poria Semen Lepidii/Descurainiae Fructus Aurantii Semen Persicae Flos Inulae Flos Carthami	**Astragalus Root** **Chinese Angelica Root** Honeysuckle Flower Red Sage Root Tuckahoe Tingli Seed Bitter Orange Peach Kernel Inula Flower Safflower	**HuangQi** **DangGui** JinYinHua DanShen FuLing TingLiZi ZhiKe TaoRen XuanFuHua HongHua
Sun ZT 2008 (Sun et al., 2008)	YiQiHuoXueSanJie Basic Prescription	**Radix Astragali** **Radix Angelicae Sinensis** Rhizoma Curcumae (Zedoariae) Radix Codonopsis Bulbus Fritillariae Cirrhosae Radix Scutellariae Radix Curcumae	**Astragalus Root** **Chinese Angelica Root** Curcuma Rhizome Codonopsis Root Fritillaria Bulb Baical Skullcap Root Curcuma Tuber	**HuangQi** **DangGui** EZhu DangShen ChuanBeiMu HuangQin YuJin
Dong H 2010 (Dong, 2010)	KangXianShuFei Granules	**Radix Astragali** **Radix Angelicae Sinensis** Radix Codonopsis Radix Scutellariae Radix Salviae Miltiorrhizae Radix Adenophorae/Glehniae Radix Paeoniae Alba Semen Lepidii/Descurainiae Herba Houttuyniae Rhizoma Pinelliae Semen Armeniacae Fructus Trichosanthis	**Astragalus Root** **Chinese Angelica Root** Codonopsis Root Baical Skullcap Root Red Sage Root Four Leaf Lady-Bell Root White Peony Root Tingli Seed Houttuynia Pinellia Rhizome Bitter Apricot Kernel Trichosanthes Fruit	**HuangQi** **DangGui** DangShen HuangQin DanShen ShaShen BaiShao TingLiZi YuXingCao BanXia XingRen GuaLou
Yang ZJ 2010 (Yang, 2010)	YiQiYangYin Prescription	**Radix Astragali** **Radix Angelicae Sinensis** Rhizoma Atractylodis Macrocephalae Radix Saposhnikoviae Radix Pseudostellariae Radix Ophiopogonis Radix Platycodi Semen Armeniacae Bulbus Fritillariae Thunbergii Rhizoma Anemarrhenae Caulis Perillae Bulbus Lilii Radix Glycyrrhizae	**Astragalus Root** **Chinese Angelica Root** Atractylodis Rhizome Saposhnikoviae Root Pseudostellaria Root Ophiopogon Tuber Balloon Flower Root Bitter Apricot Kernel Zhejiang Fritillaria Bulb Anemarrhena Rhizome Perilla Stem Lily Bulb Licorice Root	**HuangQi** **DangGui** BaiZhu FangFeng TaiZiShen MaiDong JieGeng XingRen ZheBeiMu ZhiMu ZiSuGeng BaiHe GanCao
Wang F 2011 (Wang, 2011)	KangXianShuFei Chinese Medicine	**Radix Astragali** **Radix Angelicae Sinensis** Radix Codonopsis Radix Scutellariae Radix Salviae Miltiorrhizae Radix Adenophorae/Glehniae Radix Paeoniae Alba Semen Lepidii/Descurainiae Herba Houttuyniae Rhizoma Pinelliae Semen Armeniacae Fructus Trichosanthis	**Astragalus Root** **Chinese Angelica Root** Codonopsis Root Baical Skullcap Root Red Sage Root Four Leaf Lady-Bell Root White Peony Root Tingli Seed Houttuynia Pinellia Rhizome Bitter Apricot Kernel Trichosanthes Fruit	**HuangQi** **DangGui** DangShen HuangQin DanShen ShaShen BaiShao TingLiZi YuXingCao BanXia XingRen GuaLou
Chen P 2012 (Chen et al., 2012)	HuaXianPoGu Decoction	**Radix Astragali** **Radix Angelicae Sinensis** Radix Rehmanniae Herba Epimedii Fructus Forsythiae Radix Platycodi Fructus Aurantii Semen Armeniacae Fructus Schisandrae Radix Paeoniae Alba Rhizoma Pinelliae Rhizoma Arisaematis Bulbus Fritillariae Cirrhosae Pericarpium Citri Reticulatae Rhizoma Chuanxiong Semen Persicae Pheretima Poria Radix Glycyrrhizae	**Astragalus Root** **Chinese Angelica Root** Rehmannia Root Epimedium Forsythia Fruit Balloon Flower Root Bitter Orange Bitter Apricot Kernel Schisandra Fruit White Peony Root Pinellia Rhizome Arisaema Rhizome Fritillaria Bulb Tangerine Peel Szechwan Lovage Rhizome Peach Kernel Earthworm Tuckahoe Licorice Root	**HuangQi** **DangGui** DiHuang YinYangHuo LianQiao JieGeng ZhiKe XingRen WuWeiZi BaiShao BanXia TianNanXing ChuanBeiMu ChenPi ChuanXiong TaoRen DiLong FuLing GanCao
Wu HS 2012 (Wu et al., 2012)	KangYangHuaXianRuanFei Magical Prescription	**Radix Astragali** **Radix Angelicae Sinensis** Radix Scutellariae Rhizoma Atractylodis Macrocephalae Cordyceps Radix Salviae Miltiorrhizae Rhizoma Chuanxiong Hirudo Pheretima Rhizoma Polygoni Cuspidati Rhizoma Pinelliae Bulbus Fritillariae Thunbergii Radix Ginseng Herba Epimedii Fructus Ligustri Lucidi Radix Glycyrrhizae	**Astragalus Root** **Chinese Angelica Root** Baical Skullcap Root Atractylodis Rhizome Chinese Caterpillar Fungus Red Sage Root Szechwan Lovage Rhizome Leech Earthworm Bushy Knotweed Rhizome Pinellia Rhizome Zhejiang Fritillaria Bulb Ginseng Root Epimedium Glossy Privet Berry Licorice Root	**HuangQi** **DangGui** HuangQin BaiZhu DongChongXiaCao DanShen ChuanXiong ShuiZhi DiLong HuZhang BanXia ZheBeiMu RenShen YinYangHuo NvZhenZi GanCao
Meng Y 2016 (Meng et al., 2016)	YiQiYangXue Chinese Medicine Decoction	**Radix Astragali** **Radix Angelicae Sinensis** Radix Codonopsis Rhizoma Imperatae Radix Adenophorae Radix Glehniae Radix Scrophulariae Rhizoma Phragmitis Radix Paeoniae Alba Semen Armeniacae Bulbus Fritillariae Thunbergii Radix Glycyrrhizae Radix Stemonae Fructus Jujube Radix Salviae Miltiorrhizae Carapax Trionycis	**Astragalus Root** **Chinese Angelica Root** Codonopsis Root Woolly Grass Rhizome Adenophora (Nan) Glehnia Root (Bei) Scrophularia Reed Rhizome White Peony Root Bitter Apricot Kernel Zhejiang Fritillaria Bulb Licorice Root Stemona Root Jujube Berry Red Sage Root Chinese Soft-Shell Turtle Shell	**HuangQi** **DangGui** DangShen BaiMaoGen NanShaShen BeiShaShen XuanShen LuGen BaiShao XingRen ZheBeiMu GanCao BaiBu DaZao DanShen BieJia
Zhao YD 2016 (Zhao et al., 2016)	BuFeiHuoXueHuaPi Prescription	**Radix Astragali** **Radix Angelicae Sinensis** Radix Codonopsis Flos Carthami Bulbus Fritillariae Thunbergii Radix Salviae Miltiorrhizae Rhizoma Chuanxiong Bulbus Lilii Semen Ginkgo Fructus Aurantii Fructus Perillae Bulbus Allii Macrostemi	**Astragalus Root** **Chinese Angelica Root** Codonopsis Root Safflower Zhejiang Fritillaria Bulb Red Sage Root Szechwan Lovage Rhizome Lily Bulb Ginkgo Nut Bitter Orange Perilla Fruit Chinese Garlic	**HuangQi** **DangGui** DangShen HongHua ZheBeiMu DanShen ChuanXiong BaiHe BaiGuo ZhiKe SuZi XieBai
Jiang WZ 2017 (Jiang, 2017)	YiQiHuoTanZhuYu Chinese Medicine Prescription	**Radix Astragali** **Radix Angelicae Sinensis** Radix Glehniae Radix Rehmanniae Rhizoma Chuanxiong Fructus Schisandrae Radix Ophiopogonis Pericarpium Citri Reticulatae Rhizoma Pinelliae Radix Glycyrrhizae	**Astragalus Root** **Chinese Angelica Root** Glehnia Root (Bei) Rehmannia Root Szechwan Lovage Rhizome Schisandra Fruit Ophiopogon Tuber Tangerine Peel Pinellia Rhizome Licorice Root	**HuangQi** **DangGui** BeiShaShen DiHuang ChuanXiong WuWeiZi MaiDong ChenPi BanXia GanCao
Miao G 2018 (Miao et al., 2018)	YiQiYangYinSanJieHuaTan Prescription	**Radix Astragali** **Radix Angelicae Sinensis** Radix Ophiopogonis Radix Glehniae Semen Persicae Radix Codonopsis Pseudobulbus Cremastrae/Pleiones Rhizoma Curcumae (Zedoariae) Rhizoma Sparganii Radix Salviae Miltiorrhizae	**Astragalus Root** **Chinese Angelica Root** Ophiopogon Tuber Glehnia Root (Bei) Peach Kernel Codonopsis Root Cremastra/Pleione Curcuma Rhizome Burr-Reed Rhizome Red Sage Root	**HuangQi** **DangGui** MaiDong BeiShaShen TaoRen DangShen ShanCiGu EZhu SanLeng DanShen
Yang QM 2018 (Yang, 2018)	YiQiHuoTanZhuYu Decoction	**Radix Astragali** **Radix Angelicae Sinensis** Radix Glehniae Radix Rehmanniae Rhizoma Chuanxiong Radix Ophiopogonis Fructus Schisandrae Pericarpium Citri Reticulatae Rhizoma Pinelliae Radix Glycyrrhizae	**Astragalus Root** **Chinese Angelica Root** Glehnia Root (Bei) Rehmannia Root Szechwan Lovage Rhizome Ophiopogon Tuber Schisandra Fruit Tangerine Peel Pinellia Rhizome Licorice Root	**HuangQi** **DangGui** BeiShaShen DiHuang ChuanXiong MaiDong WuWeiZi ChenPi BanXia GanCao
Ma Q 2018 (Ma, 2018)	BuYangHuanWu Decoction and LiuJunZi Decoction	**Radix Astragali** **Radix Angelicae Sinensis** Radix Paeoniae Rubra Rhizoma Chuanxiong Pheretima Rhizoma Atractylodis Macrocephalae Radix Saposhnikoviae Poria DangShen Pericarpium Citri Reticulatae Rhizoma Pinelliae Radix Glycyrrhizae	**Astragalus Root** **Chinese Angelica Root** Red Peony Root Szechwan Lovage Rhizome Earthworm Atractylodis Rhizome Saposhnikoviae Root Tuckahoe Codonopsis Root Tangerine Peel Pinellia Rhizome Licorice Root	**HuangQi** **DangGui** ChiShao ChuanXiong DiLong BaiZhu FangFeng FuLing DangShen ChenPi BanXia GanCao
Deng F 2018 (Deng and Wang, 2018)	HuangQiTaoHong Decoction	**Radix Astragali** **Radix Angelicae Sinensis** Radix Salviae Miltiorrhizae Rhizoma Chuanxiong Semen Persicae Flos Carthami	**Astragalus Root** **Chinese Angelica Root** Red Sage Root Szechwan Lovage Rhizome Peach Kernel Safflower	**HuangQi** **DangGui** DanShen ChuanXiong TaoRen HongHua
Guo SJ 2019 (Guo et al., 2019)	QiZhuKangXian Granules	**Radix Astragali** **Radix Angelicae Sinensis** Rhizoma Curcumae (Zedoariae) Fructus Corni Radix Asteris Bulbus Fritillariae Thunbergii Radix Scutellariae Radix Glycyrrhizae	**Astragalus Root** **Chinese Angelica Root** Curcuma Rhizome Asiatic Cornelian Cherry Fruit Tatarian Aster Root Zhejiang Fritillaria Bulb Baical Skullcap Root Licorice Root	**HuangQi** **DangGui** EZhu ShanZhuYu ZiWan ZheBeiMu HuangQin GanCao
Peng YF 2019 (Peng, 2019)	QiGui Prescription	**Radix Astragali** **Radix Angelicae Sinensis**	**Astragalus Root** **Chinese Angelica Root**	**HuangQi** **DangGui**

**Table 3:** Quality control of TCM prescriptions.

Study year[ref]	TCM prescriptions	Source	Species, concentration	Quality control reported	Chemical analysis reported
Sun XS 2005 (Sun, 2005)	QiHong Decoction	East Hospital of Beijing University of Chinese Medicine, Dongzhimen Hospital	Radix Astragali, 30g Radix Angelicae Sinensis, 15g et al	Prepared according to Chinese pharmacopeia	Based on previous HPLC research
Wei GS 2007 (Wei and Qiang, 2007)	TongFeiHuoXue Decoction	Affiliated Hospital of Shaanxi College of TCM	Radix Astragali, 30g Radix Angelicae Sinensis, 12g et al	Prepared according to Chinese pharmacopeia	Based on previous HPLC research
Sun ZT 2008 (Sun et al., 2008)	YiQiHuoXueSanJie Basic Prescription	The Second Hospital Affiliated to the Tianjin University of TCM	Radix Astragali, 20g Radix Angelicae Sinensis, 15g et al	Prepared according to Chinese pharmacopeia	Based on previous HPLC research
Dong H 2010 (Dong, 2010)	KangXianShuFei Granules	Tai'an TCM Hospital	Radix Astragali, 10g Radix Angelicae Sinensis, 10g et al	Prepared according to Chinese pharmacopeia	Based on previous HPLC research
Yang ZJ 2010 (Yang, 2010)	YiQiYangYin Prescription	QianFoshan Hospital of Shandong Province	Radix Astragali, n.r. Radix Angelicae Sinensis, n.r. et al	n.r.	Based on previous HPLC research
Wang F 2011 (Wang, 2011)	KangXianShuFei Chinese Medicine	First affiliated Hospital of Guangzhou Medical College, Guangdong Province	Radix Astragali, 30g Radix Angelicae Sinensis, 15g et al	Prepared according to Chinese pharmacopeia	Based on previous HPLC research
Chen P 2012 (Chen et al., 2012)	HuaXianPoGu Decoction	The first affiliated Hospital of Guangxi University of TCM	Radix Astragali, n.r. Radix Angelicae Sinensis, n.r. et al	n.r.	Based on previous HPLC research
Wu HS 2012 (Wu et al., 2012)	KangYangHuaXianRuanFei Magical Prescription	Jiuquan people's Hospital	Radix Astragali, 18g Radix Angelicae Sinensis, 9g et al	Prepared according to Chinese pharmacopeia	Based on previous HPLC research
Meng Y 2016 (Meng et al., 2016)	YiQiYangXue Chinese Medicine Decoction	Henan traditional Chinese Medicine Hospital	Radix Astragali, 15-60g Radix Angelicae Sinensis, 9g et al	Prepared according to Chinese pharmacopeia	Based on previous HPLC research
Zhao YD 2016 (Zhao et al., 2016)	BuFeiHuoXueHuaPi Prescription	The first affiliated Hospital of Dalian Medical University	Radix Astragali, 30g Radix Angelicae Sinensis, 10g et al	Prepared according to Chinese pharmacopeia	Based on previous HPLC research
Jiang WZ 2017 (Jiang, 2017)	YiQiHuoTanZhuYu Chinese Medicine Prescription	Weifang traditional Chinese Medicine Hospital	Radix Astragali, 20g Radix Angelicae Sinensis, 10g et al	Prepared according to Chinese pharmacopeia	Based on previous HPLC research
Miao G 2018 (Miao et al., 2018)	YiQiYangYinSanJieHuaTan Prescription	Traditional Chinese Medicine Hospital of Luoding City, Guangdong Province	Radix Astragali, 15g Radix Angelicae Sinensis, 15g et al	Prepared according to Chinese pharmacopeia	Based on previous HPLC research
Yang QM 2018 (Yang, 2018)	YiQiHuoTanZhuYu Decoction	The first affiliated Hospital of Henan University of traditional Chinese Medicine	Radix Astragali, 15g Radix Angelicae Sinensis, 15g et al	Prepared according to Chinese pharmacopeia	Based on previous HPLC research
Ma Q 2018 (Ma, 2018)	BuYangHuanWu Decoction and LiuJunZi Decoction	Affiliated Hospital of Gansu University of traditional Chinese Medicine	Radix Astragali, 30g Radix Angelicae Sinensis, 15g et al	Prepared according to Chinese pharmacopeia	Based on previous HPLC research
Deng F 2018 (Deng and Wang, 2018)	HuangQiTaoHong Decoction	People's Hospital of Hanchuan City, Hubei Province	Radix Astragali, 30g Radix Angelicae Sinensis, 15g et al	Prepared according to Chinese pharmacopeia	Based on previous HPLC research
Guo SJ 2019 (Guo et al., 2019)	QiZhuKangXian Granules	The second affiliated Hospital of Tianjin University of traditional Chinese Medicine	Radix Astragali Radix Angelicae Sinensis et al	Prepared according to Chinese pharmacopeia Batch number: 20130708, 20150315	Based on previous HPLC research
Peng YF 2019 (Peng, 2019)	QiGui Prescription	Central South Hospital of Wuhan University	Radix Astragali, 30g Radix Angelicae Sinensis, 6g	Prepared according to Chinese pharmacopeia	Based on previous HPLC research

TCM, traditional Chinese medicine; HPLC, high-performance liquid chromatography; n.r., not reported.

### Methodological Quality

Five RCTs (Sun et al., 2008; Ma, 2018; Miao et al., 2018; Guo et al., 2019; Peng, 2019) employed adequate methods of random sequence generation; one RCT (Guo et al., 2019) introduced allocation concealment; one RCT (Guo et al., 2019) introduced blindness and used placebo, one RCT (Yang, 2018) used double blindness, but did not describe it specifically; two RCTs (Wang, 2011; Ma, 2018) had inaccurate outcome data; and all studies were unable to know if there were selective reports (**Figures S1** and **S2**, **Table 4**).

**Table 4:** Risk of bias and quality of included RCTs.

Study year[ref]	Random sequence generation	Allocation concealment	Blinding of patient	Blinding of assessor	Incomplete outcome data	Selective reporting	Other bias	Jadad score
Sun XS 2005 (Sun, 2005)	U	U	H	H	L	U	L	2
Wei GS 2007 (Wei and Qiang, 2007)	U	U	H	H	L	U	L	1
Sun ZT 2008 (Sun et al., 2008)	L	U	H	H	L	U	L	3
Dong H 2010 (Dong, 2010)	U	U	H	H	L	U	L	1
Yang ZJ 2010 (Yang, 2010)	U	U	H	H	L	U	L	2
Wang F 2011 (Wang, 2011)	U	U	H	H	H	U	H	1
Chen P 2012 (Chen et al., 2012)	U	U	H	H	L	U	L	1
Wu HS 2012 (Wu et al., 2012)	U	U	H	H	L	U	L	1
Meng Y 2016 (Meng et al., 2016)	U	U	H	H	L	U	L	1
Zhao YD 2016 (Zhao et al., 2016)	U	U	H	H	L	U	L	1
Jiang WZ 2017 (Jiang, 2017)	U	U	H	H	L	U	L	1
Miao G 2018 (Miao et al., 2018)	L	U	H	H	L	U	L	2
Yang QM 2018 (Yang, 2018)	U	U	U	U	L	U	L	2
Ma Q 2018 (Ma, 2018)	L	U	H	H	H	U	H	3
Deng F 2018 (Deng and Wang, 2018)	U	U	H	H	L	U	L	1
Guo SJ 2019 (Guo et al., 2019)	L	L	L	L	L	U	L	5
Peng YF 2019 (Peng, 2019)	L	U	H	H	L	U	L	3

RCT, randomized controlled trial; L, low risk of bias; H, high risk of bias; U, Unclear (uncertain risk of bias).

The Jadad rating score was assigned from 1 to 5 points. Most studies had poor quality. The Jadad rating score was 5 points in one RCT (Guo et al., 2019), 3 points in three RCTs (Sun et al., 2008; Ma, 2018; Peng, 2019), 2 points in four RCTs (Sun, 2005; Yang, 2010; Miao et al., 2018; Yang, 2018; Guo et al., 2019) and 1 point in the other nine RCTs (Wei and Qiang, 2007; Dong, 2010; Wang, 2011; Chen et al., 2012; Wu et al., 2012; Meng et al., 2016; Zhao et al., 2016; Jiang, 2017; Deng and Wang, 2018) (**Table 4**).

### Outcomes

Fifteen RCTs (Sun, 2005; Wei and Qiang, 2007; Sun et al., 2008; Dong, 2010; Yang, 2010; Wang, 2011; Chen et al., 2012; Meng et al., 2016; Zhao et al., 2016; Jiang, 2017; Deng and Wang, 2018; Ma, 2018; Yang, 2018; Guo et al., 2019; Peng, 2019) compared the total effective rate of clinical effect and three RCTs (Yang, 2010; Ma, 2018; Guo et al., 2019) compared the TCM syndrome effective rate of clinical effect.

Two RCTs (Zhao et al., 2016; Guo et al., 2019) compared force vital capacity (FVC), five RCTs (Dong, 2010; Wu et al., 2012; Meng et al., 2016; Deng and Wang, 2018; Guo et al., 2019) compared FVC% predicted (FVC% pred) of lung function, one RCTs (Ma, 2018) compared total lung capacity (TLC), two RCTs (Sun, 2005; Chen et al., 2012) compared TLC% predicted (TLC% pred), four RCTs (Wei and Qiang, 2007; Zhao et al., 2016; Ma, 2018; Guo et al., 2019) compared carbon monoxide diffusing capacity (DLCO), seven RCTs (Sun, 2005; Dong, 2010; Chen et al., 2012; Wu et al., 2012; Deng and Wang, 2018; Guo et al., 2019; Peng, 2019) compared DLCO% predicted (DLCO% pred), three RCTs (Wei and Qiang, 2007; Jiang, 2017; Ma, 2018) compared vital capacity (VC) of lung function, two RCTs (Chen et al., 2012; Peng, 2019) compared VC% predicted (VC% pred), one RCTs (Zhao et al., 2016) compared forced expiratory volume in one second (FEV1), two RCTs (Dong, 2010; Meng et al., 2016) compared FEV1% predicted (FEV1% pred), two RCTs (Zhao et al., 2016; Miao et al., 2018) compared FEV1/FVC and one RCTs (Guo et al., 2019) compared ΔFVC.

Five RCTs (Meng et al., 2016; Jiang, 2017; Miao et al., 2018; Yang, 2018; Peng, 2019) compared six minute walking distance (6MWD), four RCTs (Sun et al., 2008; Wu et al., 2012; Guo et al., 2019; Peng, 2019) compared total score of St. George's Respiratory Questionnaire (SGRQ) score, three RCTs (Sun et al., 2008; Wu et al., 2012; Guo et al., 2019; Peng, 2019) compared symptoms score of SGRQ score, three RCTs (Sun et al., 2008; Wu et al., 2012; Guo et al., 2019) compared activity limitation score of SGRQ scores and three RCTs (Sun et al., 2008; Wu et al., 2012; Guo et al., 2019) compared impact score of SGRQ score and two RCTs (Meng et al., 2016; Miao et al., 2018) compared Borg scale questionnaire scores.

Ten RCTs (Sun, 2005; Wei and Qiang, 2007; Dong, 2010; Yang, 2010; Wang, 2011; Chen et al., 2012; Wu et al., 2012; Jiang, 2017; Deng and Wang, 2018; Peng, 2019) compared arterial oxygen partial pressure (PaO_2_) of arterial blood gas, four RCTs (Meng et al., 2016; Ma, 2018; Miao et al., 2018; Peng, 2019) compared arterial oxygen saturation (SaO_2_) of arterial blood gas and one RCT (Meng et al., 2016; Ma, 2018; Miao et al., 2018; Peng, 2019) compared arterial carbon dioxide partial pressure (PaCO_2_) of arterial blood gas. The arterial blood gas data in one RCT (Wang, 2011) were inaccurate.

Three RCTs (Sun et al., 2008; Chen et al., 2012; Peng, 2019) compared transforming growth factor (TGF)-β1 of serum cytokines, two RCTs (Sun, 2005; Peng, 2019) compared tumor necrosis factor (TNF)-α of serum cytokines and one RCTs (Sun, 2005) compared interleukin (IL)-8 of serum cytokines.

Five RCTs (Sun, 2005; Ma, 2018; Yang, 2018; Guo et al., 2019; Peng, 2019) compared total syndrome score of TCM, five RCTs (Sun, 2005; Yang, 2010; Meng et al., 2016; Ma, 2018; Miao et al., 2018) compared cough syndrome score of TCM, five RCTs (Sun, 2005; Yang, 2010; Meng et al., 2016; Miao et al., 2018; Ma, 2018) compared wheezing syndrome score of TCM, three RCTs (Sun, 2005; Yang, 2010; Ma, 2018) compared shortness of breath syndrome score of TCM, three RCTs (Yang, 2010; Meng et al., 2016; Miao et al., 2018) compared fatigue syndrome score of TCM, two RCTs (Yang, 2010; Meng et al., 2016) compared thirst syndrome score of TCM, two RCTs (Meng et al., 2016; Miao et al., 2018) compared coated tongue syndrome score of TCM, two studies (Meng et al., 2016; Miao et al., 2018) compared pulse manifestation syndrome score of TCM, two studies compared (Sun, 2005; Ma, 2018) phlegm syndrome score of TCM, two studies (Sun, 2005; Ma, 2018) compared velcro rale syndrome score of TCM, one RCT (Sun, 2005) compared feel suffocated syndrome score of TCM, one RCT (Sun, 2005) compared chest stuffiness syndrome score of TCM, one RCT (Yang, 2010) compared anepithymia syndrome score of TCM, one RCT (Meng et al., 2016) compared sweating syndrome score of TCM, one RCT (Ma, 2018) compared cyanosis syndrome score of TCM and one RCT (Ma, 2018) compared clubbed-finger syndrome score of TCM.

Two RCTs (Dong, 2010; Ma, 2018) compared HRCT score, one RCT (Yang, 2010) compared main symptom score (dyspnea, dry cough, chest pain, breathing rate, chest rale, X ratios, lung function, pulmonary diffusion function and arterial oxygen), one RCT (Ma, 2018) compared 6MWD scores, one RCT (Deng and Wang, 2018) compared insulin like growth factors (IGF)-1 and insulin like growth factor binding protein (IGFBP)-4 of bronchoalveolar lavage fluid (BALF), one RCT (Deng and Wang, 2018) compared HRCT effective rate, one RCT (Yang, 2010) compared effective rate of quality of life, two RCTs (Sun, 2005; Yang, 2010) compared pulmonary reinfection rate, one RCT (Yang, 2010) compared antibiotic utilization rate, one RCT (Ma, 2018) compared mMRC dyspnea scale, but its dada were inaccurate.

Adverse reactions were mentioned in the seven studies (Sun, 2005; Wei and Qiang, 2007; Yang, 2010; Meng et al., 2016; Ma, 2018; Guo et al., 2019; Peng, 2019), and the other studies did not mention whether there were adverse reactions.

The main outcomes and results are outlined in **Table 5**.

**Table 5:** Main outcomes of included RCTs.

Study year[ref]	Main outcomes	Main results (Effect size)	Adverse events
Sun XS 2005 (Sun, 2005)	1) Clinical efficacy Total effective rate 2) Pulmonary function tests tests TLC% pred DLCO% pred 3) Arterial blood gas PaO_2_ 4) Serum cytokines IL-8 TNF-α 4) Syndrome score of TCM Total syndrome score Wheezing Feel suffocated Chest stuffiness Short of breath Cough Phlegm Velcro rale 5) HRCT effective rate 6) Pulmonary reinfection rate	OR, 3.60 [1.22, 10.64] MD, 2.06 [-6.85, 10.97] MD, -1.43 [-9.79, 6.93] MD, 5.66 [0.77, 10.55] MD, -3.23 [-40.15, 33.69] MD, -3.46 [-8.40, 1.48] MD, -5.70 [-9.18, -2.22] MD, -2.10 [-2.78, -1.42] MD, -1.54 [-2.37, -0.71] MD, -0.78 [-1.50, -0.06] MD, -0.22 [-0.95, 0.51] MD, -0.37 [-0.73, -0.01] MD, 0.37 [0.01, 0.73] MD, -0.89 [-1.58, -0.20] OR, 2.40 [0.58, 9.93] OR, 0.07 [0.00, 1.24]	Experimental: No adverse reactions Control: Serum transaminase elevated(n= 4)
Wei GS 2007 (Wei and Qiang, 2007)	1) Clinical efficacy Total effective rate 2) Pulmonary function tests DLCO VC 3) Arterial blood gas PaO_2_	OR, 2.07 [0.58, 7.46] MD, 0.37 [-0.73, 1.47] MD, 0.08 [-0.10, 0.26] MD, 0.37 [-3.28, 4.02]	No adverse reactions
Sun ZT 2008 (Sun et al., 2008)	1) Clinical efficacy Total effective rate 2) SGRQ score Total score Symptoms score Activity limitation score Impact score 3) serum cytokines TGF-β1	OR, 3.25 [0.52, 20.37] MD, -5.00 [-14.83, 4.83] MD, -8.00 [-16.70, 0.70] MD, -5.00 [-15.26, 5.26] MD, -7.00 [-15.23, 1.23] MD, 0.82 [-0.06, 1.70]	n.r.
Dong H 2010 (Dong, 2010)	1) Clinical efficacy Total effective rate 2) Pulmonary function tests FVC% pred DLCO% pred FEV1% pred 3) Arterial blood gas PaO_2_ 3) HRCT score	OR, 12.93 [0.69, 244.05] MD, 9.00 [4.66, 13.34] MD, 9.00 [5.85, 12.15] MD, 7.00 [3.59, 10.41] MD, 9.00 [4.89, 13.11] MD, -1.98 [-3.80, -0.16]	n.r.
Yang ZJ 2010 (Yang, 2010)	1) Clinical efficacy Total effective rate TCM syndrome effective 2) Arterial blood gas PaO_2_ 3) Syndrome score of TCM Wheezing Cough Fatigue Short of breath Anepithymia Thirst 4) Main symptom score Dyspnea Dry cough Chest pain Breathing rate Chest rale X ratios Pulmonary function tests pulmonary diffusion function Arterial oxygen 5) Effective rate of quality of life 6) Pulmonary reinfection rate 7) Antibiotic utilization rate	OR, 8.14 [0.88, 75.48] OR, 8.14 [0.88, 75.48] MD, 2.20 [0.71, 3.69] MD, -0.40 [-1.16, 0.36] MD, -1.00 [-1.56, -0.44] MD, -0.60 [-1.19, -0.01] MD, -1.00 [-1.85, -0.15] MD, -0.60 [-1.19, -0.01] MD, -1.00 [-1.67, -0.33] MD, -0.25 [-1.78, 1.28] MD, -0.80 [-1.48, -0.12] MD, -0.20 [-1.07, 0.67] MD, -0.20 [-1.17, 0.77] MD, -0.60 [-1.32, 0.12] MD, -0.50 [-2.42, 1.42] MD, -1.00 [-3.01, 1.01] MD, -0.50 [-2.11, 1.11] MD, -1.50 [-3.63, 0.63] OR, 6.33 [0.67, 60.16] OR, 0.22 [0.06, 0.86] OR, 0.22 [0.06, 0.86]	Experimental: No adverse reactions Control: venous blood glucose increased (n= 3)
Wang F 2011 (Wang, 2011)	1) Clinical efficacy Total effective rate 4) arterial blood gas PaO_2_ PaCO_2_	OR, 4.50 [0.72, 28.15] Inaccurate data Inaccurate data	n.r.
Chen P 2012 (Chen et al., 2012)	1) Clinical efficacy Total effective rate 2) Pulmonary function tests TLC% pred DLCO% pred VC% pred 3) Arterial blood gas PaO_2_ 4) Serum cytokines TGF-β1	OR, 5.41 [1.02, 28.79] MD, 7.09 [2.12, 12.06] MD, 5.74 [1.32, 10.16] MD, 5.85 [1.37, 10.33] MD, 6.51 [0.98, 12.04] MD, -0.41 [-1.50, 0.68]	n.r.
Wu HS 2012 (Wu et al., 2012)	1) Pulmonary function tests FVC% pred DLCO% pred 2) SGRQ score Total score Symptoms score Activity limitation score Impact score 3) Arterial blood gas PaO_2_	MD, 8.20 [6.34, 10.06] MD, 7.53 [4.38, 10.68] MD, -10.00 [-15.87, -4.13] MD, -9.00 [-14.12, -3.88] MD, -12.00 [-17.35, -6.65] MD, 3.00 [-2.60, 8.60] MD, 5.87 [2.18, 9.56]	n.r.
Meng Y 2016 (Meng et al., 2016)	1) Clinical efficacy Total effective rate 2) Pulmonary function tests FVC% pred FEV1% pred 3) 6MWD 5) Borg scale questionnaire 5) Arterial blood gas SaO_2_ 6) Syndrome score of TCM Wheezing Cough Sweating Fatigue Thirst Coated tongue Pulse manifestation	OR, 2.90 [0.53, 16.03] MD, 4.83 [0.59, 9.07] MD, 3.10 [-1.22, 7.42] MD, 16.27 [-45.24, 77.78] MD, -0.66 [-1.05, -0.27] MD, 1.19 [0.26, 2.12] MD, -0.97 [-1.46, -0.48] MD, -0.66 [-1.23, -0.09] MD, -0.28 [-0.79, 0.23] MD, -1.01 [-1.53, -0.49] MD, -0.68 [-1.21, -0.15] MD, -0.77 [-1.35, -0.19] MD, -0.82 [-1.29, -0.35]	Experimental: venous blood glucose increased (n= 1) Control: venous blood glucose increased (n= 2) blood pressure elevated (n= 1) Serum transaminase elevated (n= 1)
Zhao YD 2016 (Zhao et al., 2016)	1) Clinical efficacy Total effective rate 2) Pulmonary function tests FVC DLCO FEV1 FEV1/FVC	OR, 15.62 [3.46, 70.41] MD, 0.76 [0.51, 1.01] MD, 5.03 [5.00, 5.06] MD, 0.26 [0.09, 0.43] MD, 5.90 [3.47, 8.33]	n.r.
Jiang WZ 2017 (Jiang, 2017)	1) Clinical efficacy Total effective rate 2) Pulmonary function tests VC 3) 6MWD 4) Arterial blood gas PaO_2_	OR, 3.27 [1.21, 8.84] MD, 0.30 [0.27, 0.33] MD, 29.63 [27.67, 31.59] MD, 8.20 [4.44, 11.96]	n.r.
Miao G 2018 (Miao et al., 2018)	1) Pulmonary function tests FEV1/FVC 2) 6MWD 3) Borg scale questionnaire 4) Arterial blood gas SaO_2_ 5) Syndrome score of TCM Fatigue Wheezing Cough Coated tongue Pulse manifestation	MD, 5.72 [2.34, 9.10] MD, 36.11 [5.52, 66.70] MD, -1.20 [-1.39, -1.01] MD, 6.10 [4.95, 7.25] MD, -1.53 [-1.94, -1.12] MD, -0.24 [-0.73, 0.25] MD, -1.51 [-1.95, -1.07] MD, -1.35 [-1.59, -1.11] MD, -1.63 [-2.04, -1.22]	n.r.
Yang QM 2018 (Yang, 2018)	1) Clinical efficacy Total effective rate 2) 6MWD 4) Syndrome score of TCM Total syndrome score	OR, 4.86 [1.76, 13.39] MD, 29.05 [26.14, 31.96] MD, -1.78 [-2.32, -1.24]	n.r.
Ma Q 2018 (Ma, 2018)	1) Clinical efficacy Total effective rate TCM syndrome effective 2) Pulmonary function tests DLCO TLC VC 3) Arterial blood gas SaO_2_ 4) Syndrome score of TCM Total syndrome score Short of breath Wheezing Cough Phlegm Cyanosis Clubbed-finger Velcro rale 5) HRCT score 6) mMRC dyspnea scale 7) 6MWD scores	OR, 3.75 [1.31, 10.72] OR, 3.12 [1.13, 8.60] MD, 4.34 [3.15, 5.53] MD, 0.27 [-0.02, 0.56] MD, -0.07 [-0.34, 0.20] MD, -1.03 [-2.01, -0.05] MD, 3.80 [1.06, 6.54] MD, -0.33 [-0.80, 0.14] MD, -1.66 [-2.16, -1.16] MD, -0.20 [-0.53, 0.13] MD, 0.03 [-0.32, 0.38] MD, -0.91 [-1.10, -0.72] MD, -0.40 [-0.75, -0.05] MD, -0.07 [-0.45, 0.31] MD, 0.37 [-0.08, 0.82] Inaccurate data MD, 0.12 [-0.21, 0.45]	No adverse reactions
Deng F 2018 (Deng and Wang, 2018)	1) Clinical efficacy Total effective rate 2) Pulmonary function tests FVC% pred DLCO% pred 4) Arterial blood gas PaO2 5) BALF IGF-1 IGFBP-4	OR, 2.59 [1.12, 6.02] MD, 2.99 [0.23, 5.75] MD, 3.63 [1.09, 6.17] MD, 6.67 [3.72, 9.62] MD, -0.24 [-0.35, -0.13] MD, -1.32 [-1.84, -0.80]	n.r.
Guo SJ 2019 (Guo et al., 2019)	1) Clinical efficacy Total effective rate TCM syndrome effective 2) Pulmonary function tests FVC FVC% pred DLCO DLCO% pred ΔFVC 3) SGRQ scores Total scores Symptoms scores Activity limitation scores Impact scores 4) Syndrome score of TCM Total syndrome score	OR, 8.54 [3.40, 21.50] OR, 8.54 [3.40, 21.50] MD, 0.43 [0.28, 0.58] MD, 6.18 [1.41, 10.95] MD, 2.88 [2.00, 3.76] MD, 16.97 [13.44, 20.50] MD, 0.54 [0.45, 0.63] MD, -34.00 [-38.72, -29.28] MD, -25.00 [-30.26, -19.74] MD, 1.00 [-3.25, 5.25] MD, -35.00 [-37.93, -32.07] MD, -14.82 [-15.78, -13.86]	No adverse reactions
Peng YF 2019 (Peng, 2019)	1) Clinical efficacy Total effective rate 2) Pulmonary function tests DLCO% pred VC% pred 4) 6MWD 5) SGRQ scores Total scores 3) Arterial blood gas PaO_2_ SaO_2_ 6) Serum cytokines TGF-β1 TNF-α 7) Syndrome score of TCM Total syndrome score	OR, 1.66 [0.41, 6.78] MD, -4.22[-13.62, 5.18] MD, 1.15[-5.82, 8.12] MD, 32.40 [1.49, 63.31] MD, -4.08 [-6.57, -1.59] MD, 0.89 [-5.43, 7.21] MD, 2.65 [1.15, 4.15] MD, -2.60 [-4.32, -0.88] MD, -2.56 [-4.88, -0.24] MD, -2.20 [-4.33, -0.07]	Experimental: Itch of skin (n= 1) Nausea (n= 1) Control: Itch of skin (n= 2) Nausea (n= 2) Erythra (n= 1)

n.r.: not reported.

### Meta-analysis

#### Clinical Efficacy

**Figure 2:**
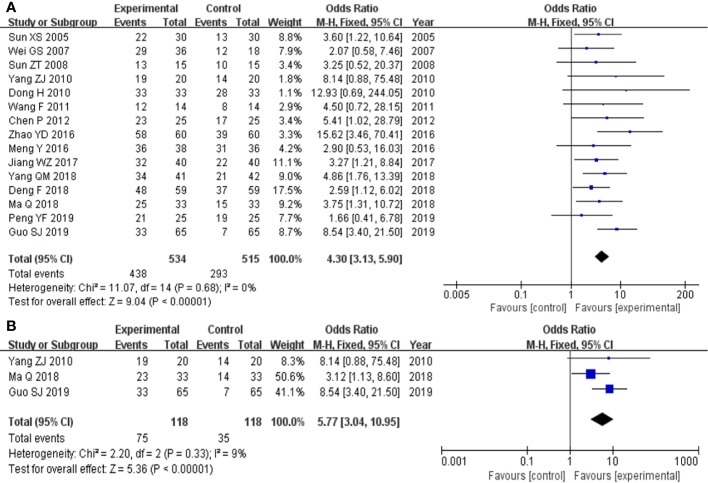
Forest plot of comparison: clinical efficacy. **(A)** Total effective rate of clinical efficacy was statistically significantly higher in experimental group than control group. **(B)** TCM syndrome effective rate of clinical efficacy was statistically significantly higher in experimental group than control group.

The 15 studies (Sun, 2005; Wei and Qiang, 2007; Sun et al., 2008; Dong, 2010; Yang, 2010; Wang, 2011; Chen et al., 2012; Meng et al., 2016; Zhao et al., 2016; Jiang, 2017; Deng and Wang, 2018; Ma, 2018; Yang, 2018; Guo et al., 2019; Peng, 2019) that compared total effective rate of clinical efficacy included a total of 1049 participants, 534 in experimental group and 515 in control group, respectively. The 15 studies had homogeneity (heterozygosity test, Chi² = 11.07, P = 0.68, I² = 0%). When the fixed effect model was used to merge OR values, the pooled OR was 4.30 (95% CI 3.31-5.90, Z = 9.04, P < 0.00001). This indicated that total effective rate of clinical efficacy was statistically significantly higher in experimental group than control group (**Figure 2A**).

The three studies (Yang, 2010; Ma, 2018; Guo et al., 2019) that compared TCM syndrome effective rate of clinical efficacy included a total of 236 participants, 118 in experimental group and 118 control group, respectively. The three studies had homogeneity (heterozygosity test, Chi² = 2.20, P = 0.33, I² = 9%). When the fixed effect model was used to merge OR values, the pooled OR was 5.77 (95% CI 3.04-10.95, Z = 5.36, P < 0.00001). This indicated that TCM syndrome effective rate of clinical efficacy was statistically significantly higher in the experimental group than in the control group (**Figure 2B**).

#### Pulmonary Function Tests

**Figure 3:**
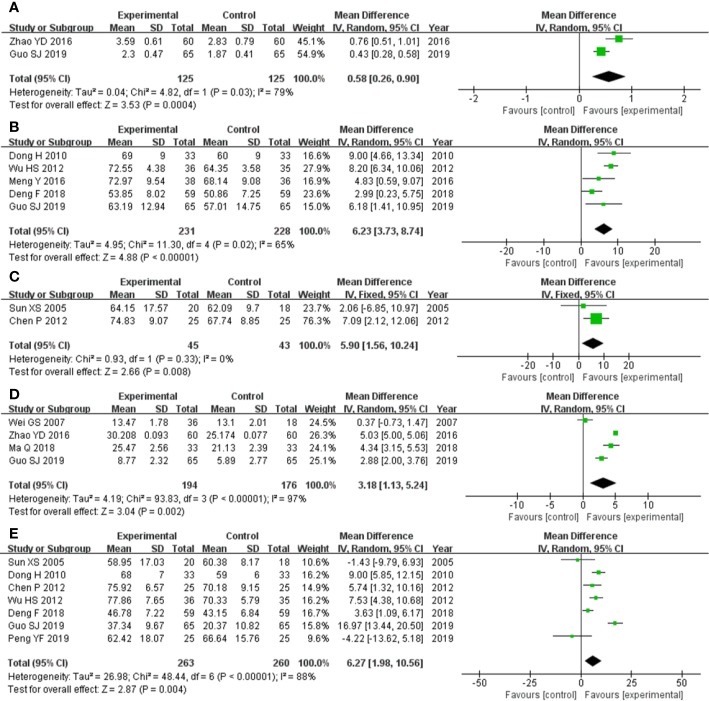
Forest plot of comparison: pulmonary function tests. **(A)** FVC was statistically significantly higher in experimental group than control group. **(B)** FVC% pred was statistically significantly higher in experimental group than control group. **(C)** TLC% pred was statistically significantly higher in experimental group than control group. **(D)** DLCO was statistically significantly higher in experimental group than control group. **(E)** DLCO% pred was statistically significantly higher in experimental group than control group.

The two studies (Zhao et al., 2016; Guo et al., 2019) that compared FVC included a total of 250 participants, 125 and 125 in experimental group and control group, respectively. The two studies had heterozygosity (heterozygosity test, Chi² = 4.82, P = 0.03, I² = 79%). When the random effect model was used to merge MD values, the pooled MD was 0.58 (95% CI 0.26-0.90, Z = 3.53, P = 0.0004). This indicated that FVC was statistically significantly higher in the experimental group than in the control group (**Figure 3A**).

The five studies (Dong, 2010; Wu et al., 2012; Meng et al., 2016; Deng and Wang, 2018; Guo et al., 2019) that compared FVC% pred included a total of 459 participants, 231 in experimental group and 228 control group, respectively. The five studies had heterozygosity (heterozygosity test, Chi² = 11.30, P = 0.02, I² = 65%). When the random effect model was used to merge MD values, the pooled MD was 6.23 (95% CI 3.73-8.74, Z = 4.88, P < 0.00001). This indicated that FVC% pred was statistically significantly higher in experimental group than control group (**Figure 3B**).

The two studies (Sun, 2005; Chen et al., 2012) that compared TLC% pred included a total of 88 participants, 45 and 43 in experimental group and control group, respectively. The two studies had homogeneity (heterozygosity test, Chi² = 0.93, P = 0.33, I² = 0%). When the fixed effect model was used to merge MD values, the pooled MD was 5.90 (95% CI 1.56-10.24, Z = 2.66, P = 0.008). This indicated that TLC% pred was statistically significantly higher in experimental group than control group (**Figure 3C**).

The four studies (Wei and Qiang, 2007; Zhao et al., 2016; Ma, 2018; Guo et al., 2019) that compared DLCO included a total of 370 participants, 194 and 176 in experimental group and control group, respectively. The four studies had heterozygosity (heterozygosity test, Chi² = 93.83, P < 0.00001, I² = 97%). When the random effect model was used to merge MD values, the pooled MD was 3.18 (95% CI 1.13-5.24, Z = 3.04, P = 0.002). This indicated that DLCO was statistically significantly higher in the experimental group than control group (**Figure 3D**).

The seven studies (Sun, 2005; Dong, 2010; Chen et al., 2012; Wu et al., 2012; Deng and Wang, 2018; Guo et al., 2019; Peng, 2019) that compared DLCO% pred included a total of 523 participants, 263 and 260 in experimental group and control group, respectively. The seven studies had heterozygosity (heterozygosity test, Chi² = 48.44, P < 0.00001, I² = 88%). When the random effect model was used to merge MD values, the pooled MD was 6.27 (95% CI 1.98-10.56, Z = 2.87, P = 0.004). This indicated that DLCO% pred was statistically significantly higher in experimental group than control group (**Figure 3E**).

#### 6MWD

**Figure 4:**
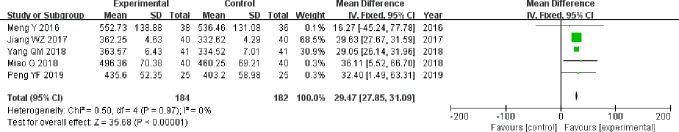
Forest plot of comparison: 6MWD. 6MWD was statistically significantly higher in experimental group than control group.

The five studies (Meng et al., 2016; Jiang, 2017; Miao et al., 2018; Yang, 2018; Peng, 2019) that compared 6MWD included a total of 366 participants, 184 and 182 in experimental group and control group, respectively. The five studies had homogeneity (heterozygosity test, Chi² = 0.50, P = 0.97, I² = 0%). When the fixed effect model was used to merge MD values, the pooled md was 29.47 (95% CI 27.85-31.09, Z = 35.68, P < 0.00001). This indicated that 6MWD was statistically significantly higher in experimental group than control group (**Figure 4**).

#### Questionnaire Score

**Figure 5:**
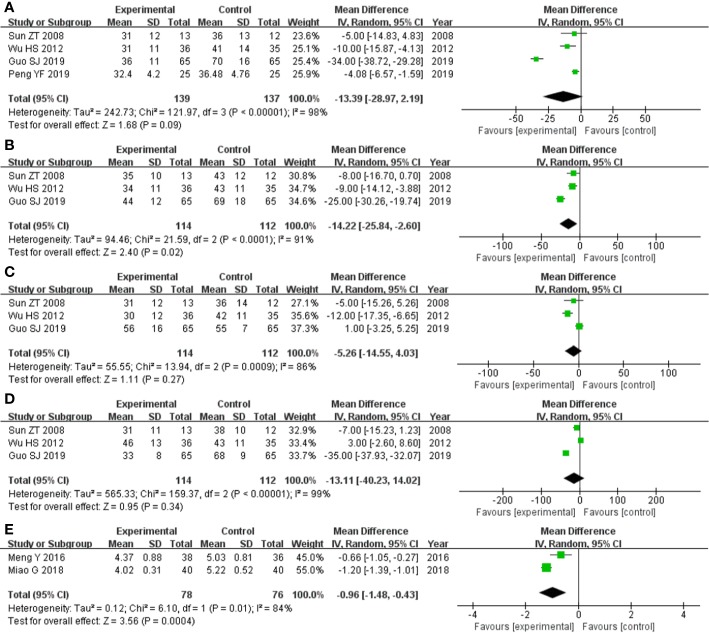
Forest plot of comparison: questionnaire score. **(A)** Comparing total score of SGRQ score, there was no significant difference between experimental group and control group. **(B)** Symptoms score of SGRQ score was statistically significantly lower in experimental group than control group. **(C)** Comparing activity limitation score of SGRQ score, there was no significant difference between experimental group and control group. **(D)** Comparing impact score of SGRQ score, there was no significant difference between experimental group and control group. **(E)** Borg scale questionnaire score was statistically significantly lower in experimental group and control group.

The four studies (Sun et al., 2008; Wu et al., 2012; Guo et al., 2019; Peng, 2019) that compared total score of SGRQ score included a total of 276 participants, 139 and 137 in experimental group and control group, respectively. The four studies had heterozygosity (heterozygosity test, Chi² = 121.97, P < 0.00001, I² = 98%). When the random effect model was used to merge MD values, the pooled MD was -13.39 [95% CI (-28.97)-(2.19), Z = 1.68, P = 0.09]. This indicated that there was no significant difference between experimental group and control group (**Figure 5A**).

The three studies (Sun et al., 2008; Wu et al., 2012; Guo et al., 2019) that compared symptoms score of SGRQ score included a total of 226 participants, 114 and 112 in experimental group and control group, respectively. The three studies had heterozygosity (heterozygosity test, Chi² = 21.59, P < 0.0001, I² = 91%). When the random effect model was used to merge MD values, the pooled MD was -14.22 [95% CI (-25.84)- (-2.60), Z = 2.40, P = 0.02]. This indicated that symptoms score of SGRQ score was statistically significantly lower in experimental group than control group (**Figure 5B**).

The three studies (Sun et al., 2008; Wu et al., 2012; Guo et al., 2019) that compared activity limitation score of SGRQ score included a total of 226 participants, 114 and 112 in experimental group and control group, respectively. The three studies had heterozygosity (heterozygosity test, Chi² = 13.94, P = 0.0009, I² = 86%). When the random effect model was used to merge MD values, the pooled MD was -5.26 [95% CI (-14.55)-(4.03), Z = 1.11, P = 0.27]. This indicated that there was no significant difference between experimental group and control group (**Figure 5C**)

The three studies (Sun et al., 2008; Wu et al., 2012; Guo et al., 2019) that compared impact score of SGRQ score included a total of 226 participants, 114 and 112 in experimental group and control group, respectively. The three studies had heterozygosity (heterozygosity test, Chi² = 159.37, P < 0.00001, I² = 99%). When the random effect model was used to merge MD values, the pooled MD was -13.11 [95% CI (-40.23)-(14.02), Z = 0.95, P = 0.34]. This indicated that there was no significant difference between experimental group and control group (**Figure 5D**).

The two studies (Meng et al., 2016; Miao et al., 2018) that compared Borg scale questionnaire score included a total of 154 participants, 78 and 76 in experimental group and control group, respectively. The two studies had heterozygosity (heterozygosity test, Chi² = 6.10, P = 0.01, I² = 84%). When the random effect model was used to merge MD values, the pooled md was -0.96 [95% CI (-1.48)-(-0.43), Z = 3.56, P = 0.0004]. This indicated that Borg scale questionnaire score was statistically significantly lower in experimental group than control group (**Figure 5E**).

#### Adverse Reactions

**Figure 6:**
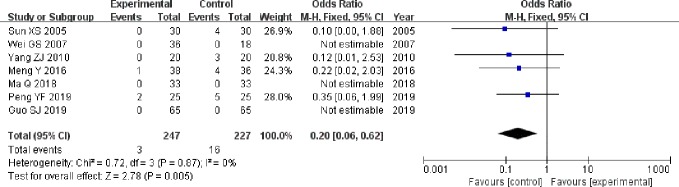
Forest plot of comparison: adverse reactions. Incidence of adverse reactions was statistically significantly lower in experimental group than control group.

The seven studies (Sun, 2005; Wei and Qiang, 2007; Yang, 2010; Meng et al., 2016; Ma, 2018; Guo et al., 2019; Peng, 2019) that compared incidence of adverse reactions included a total of 474 participants, 247 and 227 in experimental group and control group, respectively. The seven studies had homogeneity (heterozygosity test, Chi² = 0.72, P = 0.87, I² = 0%). When the fixed effect model was used to merge OR values, the pooled OR was 0.20 (95% CI 0.06-0.62, Z = 2.78, P = 0.005). This indicated that incidence of adverse reactions was statistically significantly lower in experimental group than control group (**Figure 6**).

#### Publication Bias Analysis

**Figure 7:**
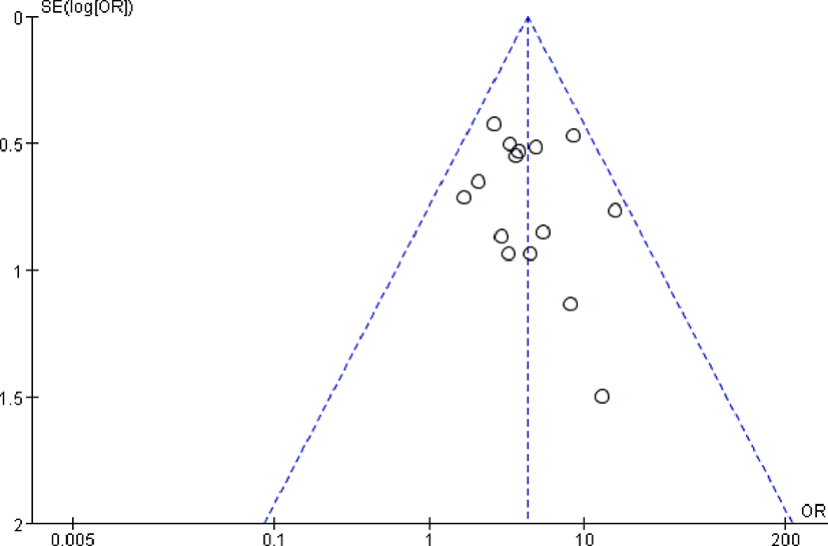
Funnel plots of total effective rate of clinical efficacy. The funnel plots showed a basically inverted and symmetrical funnel shape. The results showed that there is no obvious publication bias.

The publication bias was analyzed by funnel plots, which was drawn with the OR value of each outcome as the horizontal coordinate and SE (log [OR]) as the longitudinal coordinates. The funnel plots showed a basically inverted and symmetrical funnel shape. The results showed that there is no obvious publication bias. Funnel plots of total effective rate of clinical efficacy was shown in **Figure 7**.

## Discussion

IPF is a kind of interstitial lung disease characterized as chronic, progressive and fibrosis (Allen et al., 2020). IPF cannot be cured at present. The purpose of treatment is to delay disease progress, improve quality of life and prolong survival (Cerri et al., 2019). IPF has poor prognosis, median survival time after diagnosis is about 2 to 3 years. Pulmonary function (FVC, TLC, DLCO), PaO_2_, SGRQ score, 6MWD and cough, dyspnea symptoms are highly correlated with prognosis which are independent risk factors for IPF death (Lechtzin et al., 2013; Kim et al., 2015; Nathan et al., 2015). IPF has fewer drug options, clinical guidelines have made it clear that glucocorticoids and N-acetylcystine are not recommended or used as appropriate (Raghu et al., 2011; Group of Interstitial Lung Diseases, Respiratory Diseases Branch, Chinese Medical Association, 2016), while pirfenidone and nintedanib have certain curative effect in the treatment of IPF, but due to the high price and some side effects, they are restricted in patient use (Noble et al., 2011; Lee et al., 2013; Ryerson et al., 2019). In recent years, the position of TCM in the treatment of IPF is becoming more and more important, and the clinical research and meta-analysis have shown that the herbal medicine treating IPF could improve the clinical symptoms, delay the reduction of the lung function, improve the quality of life of the patients (Yu et al., 2016; Chen et al., 2019; Wu et al., 2019).

DangGuiBuXue Decoction is composed of RA and RAS, which has the effect of replenishing qi and generating blood. It has a history of nearly 800 years. The prescription reuses RA to replenish the qi of spleen and lung to generate the source of blood, with RAS to benefit blood and camp. Experiment studies have shown that DangGuiBuXue Decoction has a good therapeutic effect on hepatic fibrosis in rabbits (Wang and Liang, 2010), has antifibrotic effects on adriamycin-induced nephropathy in rats (Wei et al., 2012) and has antifibrosis effects on bleomycin-induced pulmonary fibrosis in rats (Gao et al., 2011; Gao et al., 2012; Zhao et al., 2015).

At present, the prescription of TCM in the treatment of IPF based on the association rules of the literature shows that the treatment of IPF with TCM is mainly related to benefiting vital energy and promoting blood circulation, among which RA and RAS are the most common herbs for invigorating qi and activating blood (Ren, 2017; Huang et al., 2018). There are many experiments on the treatment of IPF, which manifest RA and RAS can improve pulmonary fibrosis in animal model (Liu, 2009; Li et al., 2015). Our recent research shows that RA and RAS should play an effective role in the treatment of IPF through multiple targets and multiple pathways (Zhang et al., 2019).

Currently, the main study end point of IPF is the absolute value of FVC, and the secondary study end point is quality of life score and 6MWD (Noble et al., 2011; Lee et al., 2013; Ryerson et al., 2019). We analyzed these indicators primarily. In this study, the RCTs of RA and RAS in the treatment of IPF were systematically evaluated and meta-analysis was carried out. The results of meta-analysis show that total effective rate and TCM syndrome effective rate were statistically significantly higher in experimental group than control group, which suggest that RA and RAS can significantly improve the curative effect of IPF; FVC, FVC% pred, TCL% pred, DLCO and DLCO% pred, were statistically significantly higher in experimental group than control group, which suggest that RA and RAS is beneficial to pulmonary function of patients with IPF; 6MWD was statistically significantly higher in experimental group than control group and Borg scale questionnaire score was statistically significantly lower in experimental group than control group, which suggest that RA and RAS can improve exercise tolerance in patients with IPF; there was no significant difference between experimental group and control group compared total SGRQ scores, activity limitation scores and impact scores, but symptoms scores of SGRQ scores was statistically significantly lower in experimental group than control group, which suggest that RA and RAS can improve respiratory symptoms in patients with IPF, and other indexes may have more influencing factors.

We have also conducted a meta-analysis of other indicators. PaO_2_ were statistically significantly higher in experimental group than control group, which suggest that RA and RAS can improve the oxygenation in patients with IPF and there was no significant difference between experimental group than control group compared SaO_2_, which may be related to the characteristics of the oxygen dissociation curve (**Figure S3**). TNF-α was statistically significantly lower in experimental group than control group and there was no significant difference between experimental group than control group compared TGF-β1, which suggest that inhibitory inflammatory factors may play a role of RA and RAS in the treatment of IPF, but more samples are needed to further verify it (**Figure S4**).

In this systematic evaluation, the TCM syndrome effective rate and syndrome score of TCM were analyzed and made meta-analysis. The results of meta-analysis showed that TCM syndrome effective rate of clinical effect was statistically significantly higher in experimental group than control group; cough, wheezing, short of breath, fatigue, thirst, coated tongue and pulse manifestation syndrome score of TCM were statistically significantly lower in experimental group than control group. These results suggest that RA and RAS is effective in treating IPF, especially could improve the syndrome of cough, wheezing, short of breath and other syndrome which are closely related to the respiratory system (**Figure S5**).

It has been reported that TCM has potential hepatotoxicity (Teo et al., 2016; Pan et al., 2020). We also analyzed adverse reactions of include studies. Incidence of adverse reactions was statistically significantly lower in experimental group than control group, which suggest that the clinical application of RA and RAS in the treatment of IPF is safe. And there was no significant potential hepatotoxicity of RA and RAS in the treatment of IPF. Interestingly, elevated aminotransferase occurred in the control group. It is speculated that the dialectical use of TCM may reduce the toxicity and side effects of western drugs such as prednisone.

However, there are some limitations in this systematic evaluation. First of all, the study of only using RA and RAS in the treatment group is less, and we included the studies using RA and RAS as the main component in experimental group. The role of other traditional herbal medicine will have a certain impact on the results, but the role of RA and RAS as the main component is still of great significance. The next step of our research is to carry out a comparative RCT of long-term treatment of RA and RAS only in IPF. In view of the clinical particularity of TCM, and in accordance with the characteristics of real world situation, we believe that in our future read world clinical research, the experimental group should also be allowed to take other drugs, including other herbal medicine, on the basis of adhering to the rules of using RA and RAS. Secondly, some of the random methods are not clear; most of the studies do not introduce allocation concealment; most of the studies do not introduce blindness; two studies had inaccurate outcome data; and all studies were unable to know if there were selective reports. Although the quality of some research methods is low, we carefully evaluate the literature to ensure that the results are true and credible. Lastly, the treatment methods were not uniform, the dosage of RA and RAS was not the same, and the drugs in the control group were also different. Some of the research treatment cycles were short, and the safety of long-term combination of RA and RAS in the treatment of IPF could not be accurately evaluated. The existence of these biases may affect the accuracy of the research conclusions. However, our research is mainly to study the use of RA and RAS in IPF patients, so there is no special regulation on the dose and the included studies were RCTs and the diagnostic criteria was consistent, the baselines for inclusion in the literature do not differ significantly. All the prescriptions in included studies were prepared according to Chinese pharmacopeia by experts and famous old Chinese medicine practitioners and there have been many high performance liquid chromatography (HPLC) studies on RA and RAS in the past (Liu et al., 2006; Li et al., 2015; Yao et al., 2019).

## Conclusions

To sum up, RA and RAS are effective and safe in the treatment of IPF, which is beneficial to pulmonary function and exercise tolerance of these patients. Because the quality of the study is low, the quantity and sample size are small, and more high quality, multi-center, large sample RTCs are needed to obtain better evidence.

## Author Contributions

YZ conducted the database search, assessed studies for inclusion, extracted and analyzed the data, and drafted the manuscript. LG drafted the manuscript, amended English writing of this review, and revised the manuscript. QX assessed studies for inclusion, extracted the data, and arbitrated any disagreements. LT amended English writing of this review and arbitrated any disagreements. JQ conducted the database search, assessed studies for inclusion, extracted and analyzed the data, and drafted the manuscript. MC supervised YZ, LG, and JQ to perform this review and revised the manuscript.

## Funding

This study was partially supported by the National Natural Science Foundation of China (Grant 81200049 and Grant 81670059 to MC and Grant 81302768 to JQ), the Nanjing Medical Science and Technique Development Foundation (Grant QRX17005 to MC), the Nantong Science and Technology Plan Project (Grant MS12017004-2 to LT), and Research Grant of Jiangyin Hospital of Traditional Chinese Medicine to YZ.

## Conflict of Interest

The authors declare that the research was conducted in the absence of any commercial or financial relationships that could be construed as a potential conflict of interest.

## Abbreviations

IPF, idiopathic pulmonary fibrosis; RA, Radix Astragali; RAS, Radix Angelicae Sinensis; RCT, randomized controlled trial; HRCT, high-resolution CT; UIP, usual interstitial pneumonia; TCM, traditional Chinese medicine; MD, mean difference; CI, confidence interval; OR, odds ratio; CWMT, conventional western medicine treatment; FVC, force vital capacity; FVC% pred, FVC% predicted; TLC, total lung capacity; TLC% pred, TLC% predicted; DLCO, carbon monoxide diffusing capacity; DLCO% pred, DLCO% predicted; VC, vital capacity; VC% pred, VC% predicted; FEV1, forced expiratory volume in one second; FEV1% pred, FEV1% predicted; 6MWD, six minute walking distance; SGRQ, St. George's Respiratory Questionnaire; PaO_2_, arterial oxygen partial pressure; SaO_2_, arterial oxygen saturation; PaCO_2_, arterial carbon dioxide partial pressure; TGF, transforming growth factor; TNF, tumor necrosis factor; IL, interleukin; IGF, insulin like growth factors; IGFBP, insulin like growth factor binding protein; BALF, bronchoalveolar lavage fluid; HPLC, high performance liquid chromatography; n.r., not reported.
