# Supplementary material for: Radix Astragali and Radix Angelicae Sinensis in the Treatment of Idiopathic Pulmonary Fibrosis: A Systematic Review and Meta-analysis
Source: Front Pharmacol. 2020 Apr 30;11:415. doi: 10.3389/fphar.2020.00415 (PMC7203419; doi:10.3389/fphar.2020.00415)
Supplement: Supplementary file 1 [file DataSheet_1.docx]

**Supplement Figure and Figure Legends:**


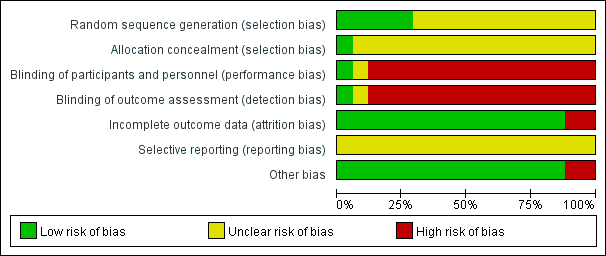


**Fig.S1.** Risk of bias graph

Review authors' judgements about each risk of bias item presented as percentages across all included studies.


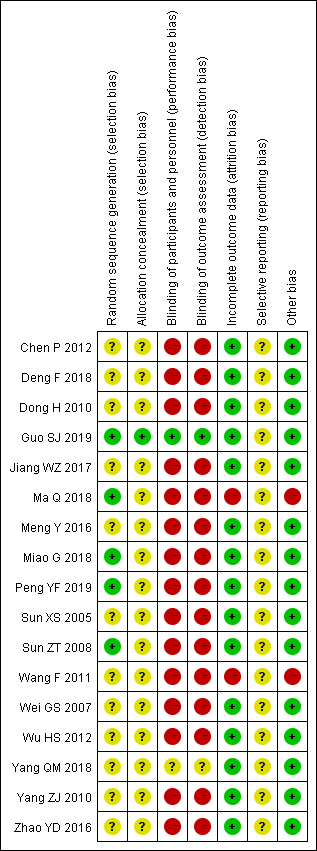


**Fig.S2.** Risk of bias summary

Review authors' judgements about each risk of bias item for each included study.

**
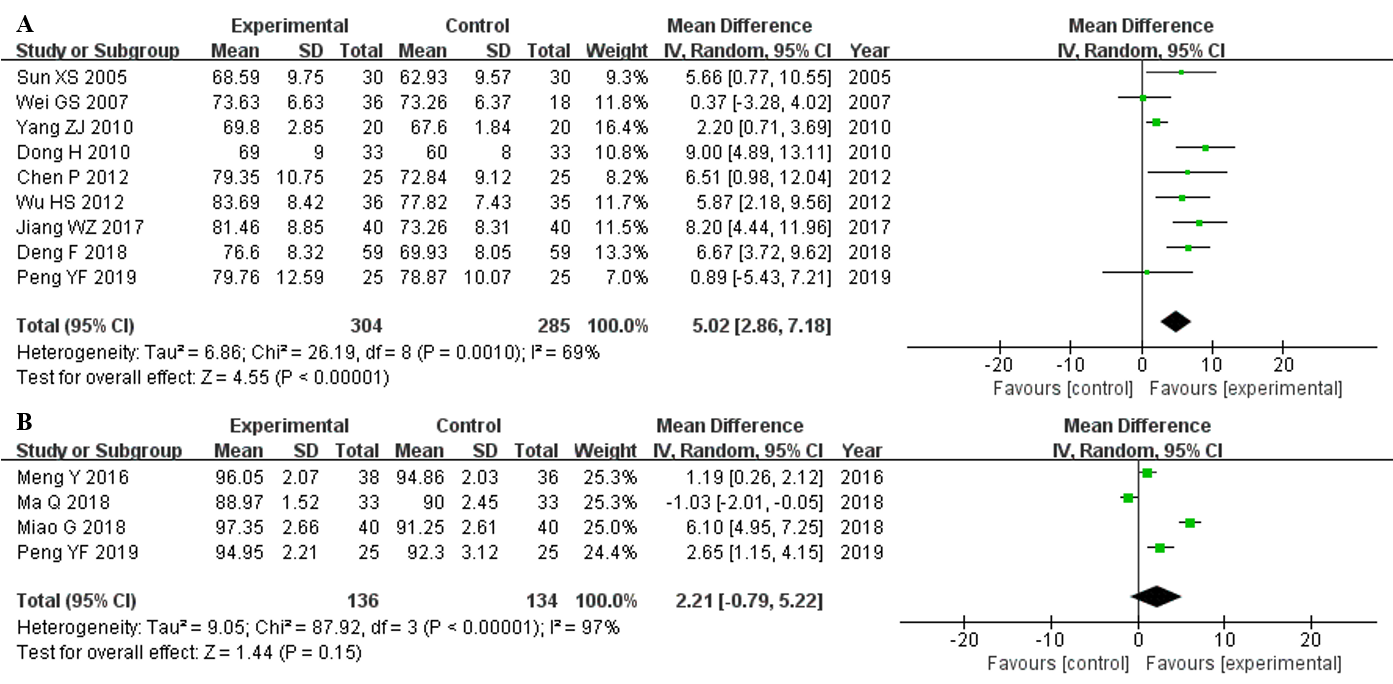
**

**Fig.S3.** Forest plot of comparison: arterial blood gas

(A) PaO_2_ of arterial blood gas was statistically significantly higher in experimental group than control group. (B) Comparing SaO_2_, there was no significant difference between experimental group than control group.


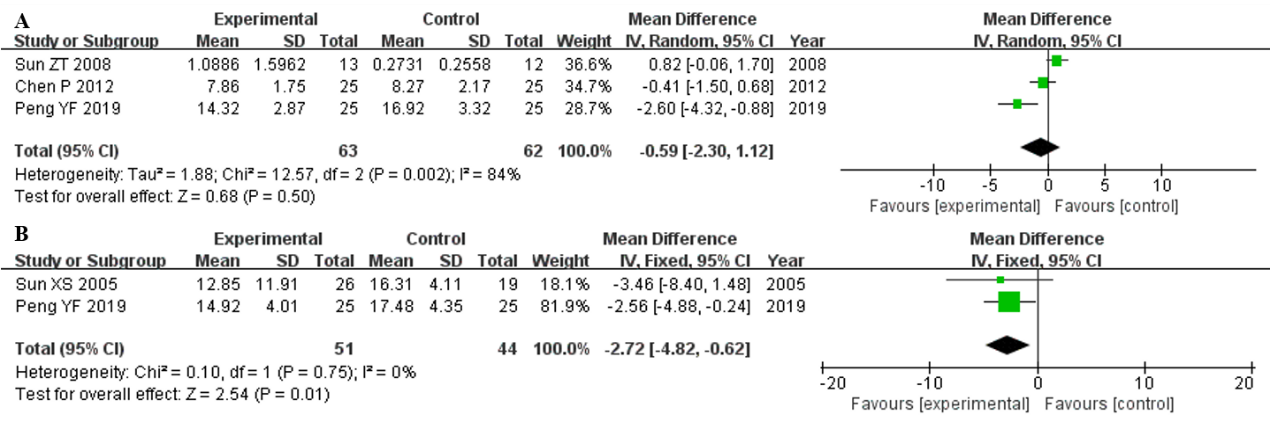


**Fig.S4.** Forest plot of comparison: serum cytokines

(A) Compared TGF-β1, there was no significant difference between experimental group than control group. (B)TNF-α was statistically significantly lower in experimental group than control group.


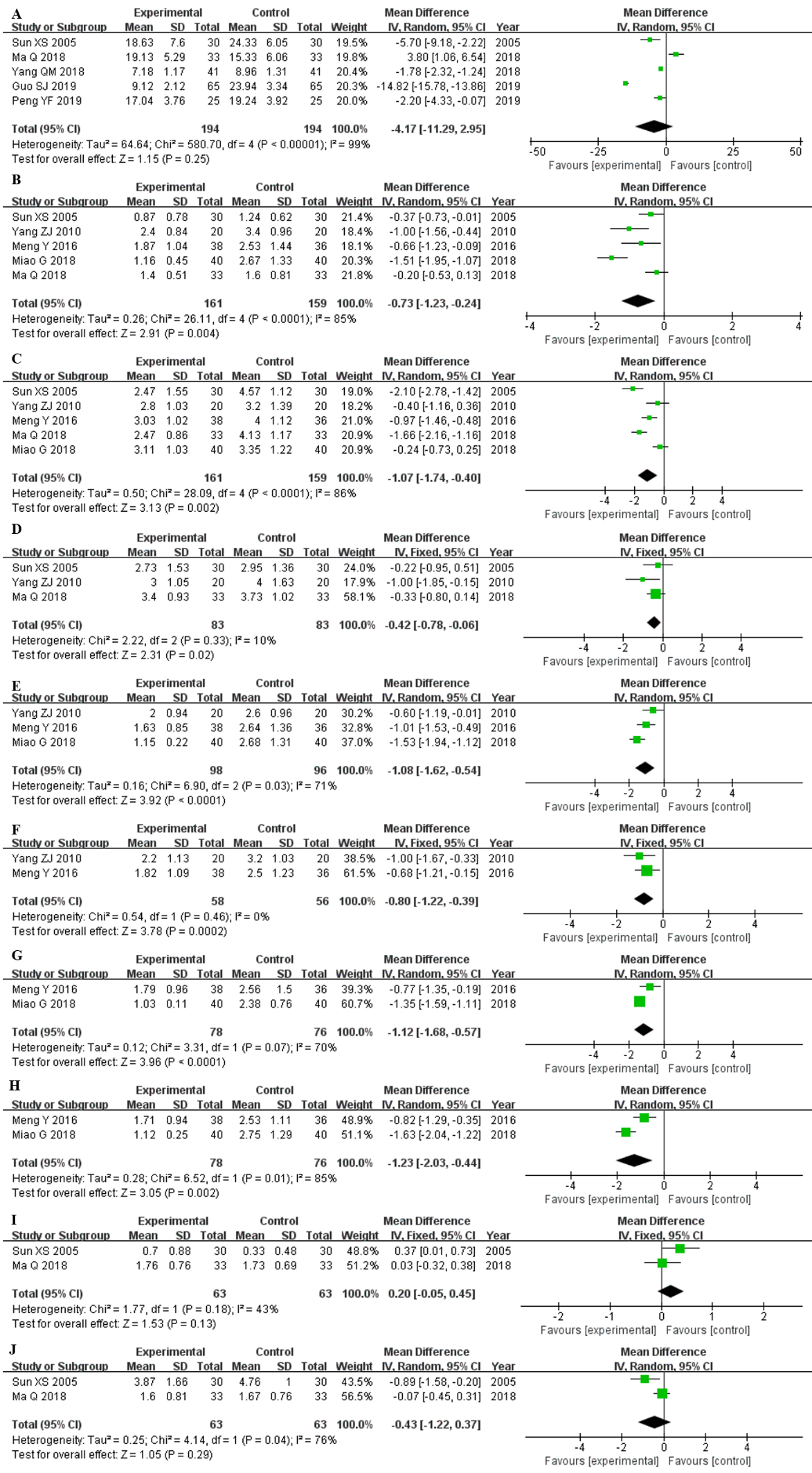


**Fig.S5.** Forest plot of comparison: syndrome score of TCM

(A) Comparing total syndrome score of TCM, there was no significant difference between experimental group than control group. (B) Cough syndrome score of TCM was statistically significantly lower in experimental group than control group. (C) Wheezing syndrome score of TCM was statistically significantly lower in experimental group than control group. (D) Short of breath syndrome score of TCM was statistically significantly lower in experimental group than control group. (E) Fatigue syndrome score of TCM was statistically significantly lower in experimental group than control group. (F) Thirst syndrome score of TCM was statistically significantly lower in experimental group than control group. (G) Coated tongue syndrome score of TCM was statistically significantly lower in experimental group than control group. (H) Pulse manifestation syndrome score of TCM was statistically significantly lower in experimental group than control group. (I) Comparing phlegm syndrome score of TCM, there was no significant difference between experimental group than control group. (J) Comparing velcro rale syndrome score of TCM, there was no significant difference between experimental group and control group.
